# Sleep spindles, tau, and neurodegeneration

**DOI:** 10.1093/sleep/zsac161

**Published:** 2022-07-07

**Authors:** Thomas C Neylan, Christine M Walsh

**Affiliations:** Department of Psychiatry and Behavioral Sciences, University of California, San Francisco, CA, USA; Veterans Affairs Medical Center, San Francisco, CA, USA; Department of Neurology, University of California, San Francisco, CA, USA; Department of Neurology, University of California, San Francisco, CA, USA

There has been a rapid growth of scientific evidence demonstrating the bidirectional role of disturbed sleep on risk and progression of neurodegenerative disease. The evidence stems from four lines of inquiry: (1) sleep disorders, particularly obstructive sleep apnea syndrome, increase risk for the development and progression of cognitive decline and dementia [1–3]; (2) sleep disruption has been demonstrated to having a causal role in the increased production of amyloid β (Aβ) and other proteins implicated in neurodegeneration [4–6]; (3) clearance of brain Aβ, tau, α-synuclein, and other proteins is facilitated by the increase in flow of interstitial fluid in the brain (e.g. glymphatic system) strongly associated with non-rapid eye movement (REM) slow-wave sleep [7–10]; and (4) focal and network issues related to the spread of misfolded proteins and site-specific vulnerability across proteinopathies [11] account for different sleep phenotypes being linked to different neurodegenerative disorders (e.g. REM behavior disorder with alpha synucleinopathy [12]; hyperarousal in progressive supranuclear palsy [13]). Given that different neurodegenerative disorders are characterized by stereotypic patterns of disease onset and spread, and given that different facets of sleep can be studied topographically, the study of local features of sleep are deepening our understanding of sleep and dementia [14].

Mander and colleagues examined the relationship of topographical sleep spindle measures to cerebrospinal fluid (CSF) measures of astrocyte and microglial activation, biomarkers for Alzheimer’s disease (AD), measures of synaptic and axonal integrity, and overnight memory consolidation in a sample of older cognitively healthy subjects at risk for the development of AD [15]. The CSF measures were obtained a couple years apart from the sleep and memory studies and the intervals between the measures were entered as covariates in linear models. Importantly, all subjects were tau pathology negative and all but one was Aβ negative by established criteria [16]. They found that age was associated with increases in three CSF markers of glial activation, glial fibrillary acidity protein (GFA), chitinase-3-like protein (YKL-40), and soluble triggering receptor expressed on myeloid cell 2 (sTREM2), two biomarkers of AD (total and phosphorylated tau), and measures of synaptic (α-synuclein [16]) and axonal integrity (neurofilament light chain [NfL]). The research team analyzed oscillatory activity across a wide range of frequencies and distribution and found that fast spindle activity (13 – <16 Hz), density, and duration declined with age predominantly in frontal and parietal sites. The notable findings from this report were that CSF measures of glial activation were associated with frontal decreases in fast spindle activity as well as tau-related AD biomarkers and measures of synaptic and axonal integrity, including neurogranin which was not associated with age. Mediation analyses showed that the effects of age on fast spindle activity were linked to glial activation, tau phosphorylation, and decline in synaptic integrity. Further, there were measurable functional consequences of reduced frontal fast spindle activity on sleep-related memory retention. The authors concluded with a hypothesis that age-related decline in memory function stems from the decline of fast spindle activity arising from increased inflammation, tau pathology, and synaptic degeneration even in the absence of clinical indicators of dementia.

The report by Mander and colleagues has many strengths. It is methodologically rigorous, focused on CSF markers instead of peripheral blood, and has appropriate statistical adjustments for the many analyses. The multilevel approach examining age, cognition, sleep spindles, glial function, CSF AD biomarkers, and measures of synaptic and axonal integrity is quite innovative and proved to be illuminating. The authors were appropriately circumspect about the strength of their findings and acknowledged that their mediation analyses have limits for making strong causal inferences. They suggested future longitudinal studies and experimental manipulations to ultimately identify the causal drivers. Surprisingly, they did not find an effect of apnea events on tau or other CSF measures as has been demonstrated in basic model systems [17]. Another caveat, which the authors acknowledge, is that this study should not be interpreted as providing evidence that inflammation and tau are key drivers of normal age-related changes to sleep. By design, the study focused on a cohort at risk for AD, and not representative for normal aging. Though this report was focused on fast spindle activity, the authors also noted that the interaction and coupling of several sleep-related oscillatory events (slow oscillations, slow-wave sleep activity, hippocampal ripples, and spindles) may prove to be the most informative for understanding sleep-related memory consolidation [14].

The presence of phosphorylated tau in neurons does not appear to explain why some neurons die and other don’t across different neurodegenerative diseases [18]. For example, nuclei in some tauopathies may be associated with a high content of phospho-tau yet are able to remain functional [11]. The important interaction of proteinopathies and inflammation has strong support; however, the interaction is quite complex and not consistently observed [19]. As an example, YKL-40 is implicated in AD, but not in dementia with Lewy bodies, Parkinson’s disease dementia, or vascular dementia [20]. YKL-40 can be pro- or anti-inflammatory depending on circadian and other contextual factors [21, 22]. Glia interactions with neurons involve more complex processes such as synaptic homeostasis and metabolic support that extend beyond than classic neuroimmune functions [23]. Thus, the contribution of pathological tau species to loss of spindle function may include other pathways involved in neuronal support outside of inflammation.

The lack of relationship of Aβ markers with measures of tau, neuronal, and axonal integrity in this report are consistent with the emerging evidence that tau can be a primary driver of neurodegeneration even in the absence of amyloid deposition [24]. However, once present, deposition of Aβ can facilitate microglial activation and colocalization with the spread of tau, in a stereotypic pattern that follows Braak stages [25], in living subjects with early evidence of AD [26]. Overall, the study from Mander et al. provides important new evidence supporting the key interaction of glial mediators with phosphorylated tau to produce neural damage.

Given that sleep is increasingly understood as a causal driver of neurodegeneration, sleep-focused interventions should be considered a potential disease modifying therapy as opposed to symptom management. Further, given the heterogeneity of different neurodegenerative disorders with respect to disease onset and pattern of progression, the study of neurodegenerative disorders as models of naturalistic lesions may deepen our understanding of basic sleep mechanisms in the human brain. Lesion studies have long led major advances in understanding basic sleep–wake biology [27–30]. One conundrum highlighted by the report by Mander and colleagues is how an oscillatory phenomena like sleep spindles that are produced by a deep-seated generator (e.g. reticular nucleus of the thalamus [31]), could present with topographical variation on the surface of the cortex in *density* of activity [32]? It would be interesting to see if spindle distribution in patients with frontotemporal dementia versus posterior cortical atrophy replicates the divergent anterior to posterior topography of spindles suggested by the underlying pathology. Human neuropathology studies probing thalamic micromorphology, topographical distribution of neurodegeneration across the cortex, and pathology in the fibers that connect the thalamus to the cortex across neurodegenerative studies will be needed to fully explain differences in rate of spindle density and distribution associated with age and disease. Outside of human neuropathology studies, the use of multidimensional studies, examining sleep, disease biomarkers, glial markers, and proxy measures of neuronal integrity, exemplified by the work of Mander and colleagues, will deepen our understanding of the relationship of sleep and neurodegeneration and potentially lead to discoveries about other basic sleep mechanisms in the human brain.
